# Implementation of a Geriatrics-Primary Care co-management model in an urban community health center

**DOI:** 10.1093/geroni/igaf122.2866

**Published:** 2025-12-31

**Authors:** Carolina Fonseca Valencia, Jeremy Whyman

**Affiliations:** Beth Israel Deaconess Medical Center, Brookline, Massachusetts, United States; Beth Israel Deaconess Medical Center, Boston, Massachusetts, United States

## Abstract

Vulnerable socioeconomically disadvantaged (SEcD) older adults represent a diverse and complex group with high levels of socioeconomic stressors, low health literacy, and chronic medical conditions. These complexities make diagnosis and management of geriatric syndromes difficult resulting in care delays, increased preventable costs, morbidity, and mortality. Geriatric co-management is characterized by geriatricians and non-geriatricians working collaboratively to detect, prevent, and manage geriatric syndromes. This model of care utilizes the comprehensive geriatrics assessment (CGA) as a primary tool, to frame the care of SEcD older adults, however, its impact in community health settings is not well known. To lessen healthcare inequities and understand the impact that specialized geriatrics care has in the health management of SEcD older adults, we have implemented a geriatric co-management model in an urban community health center. Our intent is to enhance early diagnosis and management of geriatric syndromes and support the primary care team by demonstrating feasibility of implementation; defining the specific characteristics of this population; and describing the prevalence of geriatric syndromes. In a 24-month period we have completed a total of 100 CGAs on patients between the ages of 53-100 years. Most patients are female (62%), non-English speaking (65%) with Cape Verdean, Creole and Spanish being the most prevalent languages. These patients are mostly moderately frail with average frailty index of 0.404, and the most common geriatric syndromes include cognitive impairment (MCI and dementia), malnutrition, polypharmacy, and gait disturbances.

